# Sprint skating profile of competitive male bandy players: determination of positional differences and playing level

**DOI:** 10.3389/fphys.2023.1055863

**Published:** 2023-05-26

**Authors:** Roland van den Tillaar, Haris Pojskic, Håkan Andersson

**Affiliations:** ^1^ Department of Sports Sciences, Nord University, Levanger, Norway; ^2^ Department of Sports Science, Linnaeus University, Kalmar, Sweden; ^3^ High Performance Center, Växjö, Sweden

**Keywords:** acceleration, peak velocity, anthropometrics, elite players, speed skating

## Abstract

This study aimed to compare sprint skating profile characteristics of the different playing positions of junior and senior bandy players. In total, 111 male national-level bandy players (age: 20.7 ± 5.0 years, height: 1.80 ± 0.05 m, body mass: 76.4 ± 0.4 kg, training experience: 13.8 ± 5.0 yrs) were tested on their sprint skating profile over 80 m. The main findings were that no differences between positions were found in sprint skating performance (speed and acceleration), but that elite players were in general heavier (*p* < 0.05) than junior players (80.0 ± 7.1 vs. 73.1 ± 8.1 kg), they could accelerate faster (2.96 ± 0.22 vs. 2.81 ± 0.28 m/s^2^), and they reached a higher velocity (10.83 ± 0.37 vs. 10.24 ± 0.42 m/s) earlier over 80 m than the junior players. This implies that junior level players should spend more time in power and sprint training to meet the specific demands of playing at a higher, elite level.

## 1 Introduction

Bandy is the second largest winter team sport, beaten only by ice hockey, with over 350,000 practitioners (Persson et al., 2021). It can be categorized as a large field team sport because it is played on a soccer-sized ice rink between two teams, each consisting of 11 players including a goalkeeper. All players use skates and every player, but the goalkeeper has a club. The purpose of the game is to use the club to turn a ball into the opposing team’s goal within 90 min of match time. Bandy is the forerunner of ice hockey, and there was no clear distinction between the two sports until the 1920s.

Bandy is an intermittent physically demanding sport with many defensive and offensive actions that impose a high external and internal workload load on players, with both aerobic and anaerobic bioenergetic pathways being hugely activated during a match (Blomqvist et al., 2018; Johansson et al., 2021; Persson et al., 2021). For instance, elite bandy players spend 40–80 min (i.e., 36%–71% of the playing time) in a heart rate zone between 81% and 100% of their maximum, with 15–27 min spending above their lactate threshold during a match, in which defenders had the highest scores while forwards the lowest (Blomqvist et al., 2018). A recent study has revealed that the total distance covered in bandy can vary between 21.1 ± 3.5 km and 23.2 ± 2.4 km, with mean skating velocity extending 16 km/h. Furthermore, it was found that defenders cover more distance but at a lower mean skating velocity when compared to forwards (Blomqvist et al., 2018; Persson et al., 2021).

External workload is magnified by match demands that require players to repeatedly engage in sequences of high-intensity activities, such as rapid accelerations and decelerations, and high-velocity skating that in some cases reaches 37 km/h (Blomqvist et al., 2018; Johansson et al., 2021; Persson et al., 2021). Recently, Johansson et al. (2021) reported that elite bandy players cover approximately 2.4 km very quickly and ≈600 m in sprinting skating during a match play. This is a consequence of the players covering a distance of 450 m while performing high and very high-intensity accelerations and decelerations.

It is, therefore, logical to conclude that high-velocity skating performance is one of the most important skills in bandy because it is necessary to win ball possession or to outperform an opponent. For instance, in ice hockey (which has similarities to bandy concerning the skating movement pattern) acceleration ability and velocity increase the probability of winning (Renaud et al., 2017; Douglas et al., 2019). In addition, it is shown that acceleration rates decreases from period one with period two and three (Brocherie et al., 2018; Perez et al., 2022a). Furthermore, to have better insight in sprinting performance, it is crucial to investigate playing position and level differences in bandy players. Persson et al. (2021) quantified and analyzed sport-specific demands regarding five playing positions in 10 elite bandy players. In brief, the offensive players spent significantly more time in fast (20.8 ± 5.5 vs. 13.9 ± 6.0 min), very fast (7.3 ± 2.2 vs. 4.1 ± 2.5 min), and sprint skating (1.2 ± 0.6 vs. 0.9 ± 0.8 min) than defensive players. Similar findings were reported by Douglas et al. (2019), who found that defensive players in ice hockey spent less time in high-intensity skating and glided for a shorter time when compared to offensive players (e.g., forwards). However, to our knowledge, there is no research of the different playing level requirements in bandy (e.g., physiological, anthropometrics, and sport-specific requirements). In addition, there are no published studies that have used an experimental approach (i.e., on-ice measurements) to examine sprinting profile in elite bandy players. The previously mentioned studies only included 10 professional players, which could potentially reveal the individual’s characteristics rather than actual demands of the game (Blomqvist et al., 2018; Johansson et al., 2021; Persson et al., 2021).

This study aims to compare skating sprint profile of junior and senior elite bandy players of different positions (defender, midfielders and forwards). We hypothesize that sprint skating profile of bandy players would vary according to their different playing positions and playing levels. Based on the previous studies, it is reasonable to believe that offensive bandy players (forwards) and senior players will have higher skating velocity than other playing positions and junior players, respectively. If significant differences exist among playing positions and levels, then it may provide an insight into the sprint skating qualities that are important for success in that position and level. It will also provide a greater understanding of the factors that limit the performance of these players. In addition, this information can be used to provide appropriately structured training programs for each playing position and playing level.

## 2 Methods

### 2.1 Participants

A group of 111 male bandy players (age: 20.7 ± 5.0 years, height: 1.80 ± 0.05 m, body mass: 76.4 ± 0.4 kg, training experience: 13.8 ± 5.0 yrs) participated in the study. The players were categorized according to level (47 elite and 64 junior elite players) and playing position (43 defenders, 38 midfielders and 30 forwards). At the time of the study, they had to play at least at national level. No participants had reported a history of neuromuscular disease or injuries in the previous 6 months. Two days prior to the experimental visits, the subjects were asked to avoid sleep deprivation, to refrain from high-intensity training, and to avoid tobacco, alcohol, and caffeine. They trained 10–12 h a week (five to six sessions of 2 h each) on the ice to improve technical and tactical skills, and 3–4 h a week (two sessions of 1.5 to 2 h each) off the ice in the gym to improve their strength and power. All the participants were fully informed verbally and in writing about the procedures and they signed a written consent (which was also signed by their parents when under 18 years of age) before participation. This study was part of a larger project where also the same measurements were performed on female bandy players. For statistical and transparency reasons (preforming 4-way ANOVAs) the collected data was separated in two data sets and presented separately. Data from the female bandy players is already published by van den Tillaar et al. (2023). The study was conducted following the latest revision of the Declaration of Helsinki and current ethical regulations for research and was approved by the Swedish Ethical Review Authority (No: 2022-01550-01).

### 2.2 Procedure

All testing was carried out in the period between January and March 2022. All tests were conducted in an indoor facility to eliminate the effect of the weather conditions on the results. Before testing the skating performance, anthropometric variables of height and body mass were measured in each subject. Thereafter, three participants at a time performed their own preferred warm-up for 15 min to be ready for testing in the 80 m linear sprint skating. The participants wore their regular training gear during warm-up and testing. After their warm-up, the participants sat down on a chair where the testing procedure was described to them. After 5 min of rest, each participant performed two maximal 80 m sprint skating attempts with their club in their hand (to have the same condition as in competition), starting with the club behind the starting line.

Pairs of photocells (Egotest AS, Porsgrunn, Norway) were put at the start and at 10, 20, 30, 40, 50, 60, 70 and 80 m, at 1.2 m height to measure the times at these distances and to calculate the average velocities between these different distances. Average acceleration per 10 m was calculated by the using difference of velocity per 10 m divided by the times per 10 m. The participants started 0.5 m behind the starting line and had to skate for 90 m to avoid quitting too early before the finish line. All the participants were encouraged to skate as quickly as possible over 90 m in a straight line. The total ice rink size was 110 m long. Each player repeated the same procedure for two attempts and only the best time taken to cover 80 m distance in the sprint skating test was used in data analysis. A rest period of 5 min was provided between attempts.

### 2.3 Statistical analysis

Data is expressed as mean ± SD. To compare the anthropometric and sprint skating profile of the different playing positions and levels, a 2 (level: junior and elite) x 3 (position: defenders, midfielders and forwards) analysis of variance (ANOVA) was used. In addition, on the velocity and acceleration per 10 m distance, an 8 (velocity/acceleration per 10 m distance: repeated measures) x 2 (level) x 3 (position) ANOVA was used. Where significant differences were found, a Holm-Bonferroni probability adjustment *post hoc* test was used to determine the source(s) of those differences. Effect size was evaluated with η_p_^2^ (partial eta-squared), where 0.01< η_p_^2^<0.06 represents a small effect, 0.06< η_p_^2^<0.14 represents a medium effect, and a large effect when η_p_^2^>0.14. All analyses were performed using SPSS Version 25.0. Statistical significance was set at *p* < 0.05.

## 3 Results

No significant effect for playing positions and interaction (playing position x level) effects were found for body mass and height, and sprint skating times (F ≤ 2.27, *p* ≥ 0.11, η_p_^2^ ≤ 0.04). A significant effect was found for playing level on body mass and all sprint times (F ≥ 7.2, *p* ≤ 0.008, η_p_^2^≥0.07, Table 1), in which the elite players were heavier and faster at all distances than the junior elite players.

**TABLE 1:** Mean (±SD) anthropometrics and sprint times at the different distances for each playing position and level.

Parameter	Position					
	Elite			Junior elite		
	Defence	Midfield	Forward	Defence	Midfield	Forward
Number (n)	16	17	15	27	21	15
Height (m)	1.82 ± 0.06	1.82 ± 0.05	1.79 ± 0.06	1.80 ± 0.05	1.80 ± 0.05	1.78 ± 0.05
Body mass (kg)	81.0 ± 6.1^a^	82.6 ± 6.5^a^	78.8 ± 6.1^a^	74.5 ± 8.5	73.0 ± 5.4	70.4 ± 8.7
*Sprint times*						
10 m (s)	1.83 ± 0.09	1.86 ± 0.06^a^	1.83 ± 0.08	1.89 ± 0.10	1.90 ± 0.11	1.86 ± 0.07
20 m (s)	3.10 ± 0.10^a^	3.13 ± 0.08^a^	3.08 ± 0.12^a^	3.21 ± 0.13	3.24 ± 0.12	3.20 ± 0.08
30 m (s)	4.26 ± 0.09^a^	4.26 ± 0.07^a^	4.17 ± 0.09^a^	4.39 ± 0.17	4.41 ± 0.17	4.37 ± 0.09
40 m (s)	5.28 ± 0.13^a^	5.31 ± 0.12^a^	5.24 ± 0.18^a^	5.49 ± 0.21	5.50 ± 0.21	5.46 ± 0.11
50 m (s)	6.29 ± 0.15^a^	6.32 ± 0.14^a^	6.25 ± 0.20^a^	6.55 ± 0.24	6.56 ± 0.23	6.52 ± 0.14
60 m (s)	7.27 ± 0.18^a^	7.29 ± 0.17^a^	7.21 ± 0.23^a^	7.57 ± 0.29	7.56 ± 0.27	7.53 ± 0.16
70 m (s)	8.22 ± 0.20^a^	8.24 ± 0.19^a^	8.15 ± 0.26^a^	8.57 ± 0.33	8.55 ± 0.29	8.52 ± 0.19
80 m (s)	9.16 ± 0.22^a^	9.19 ± 0.21^a^	9.09 ± 0.30^a^	9.56 ± 0.37	9.52 ± 0.33	9.50 ± 0.21

^a^ Indicates a significant difference between the elite and junior group for this position on a *p* < 0.05 level.

**FIGURE 1:**
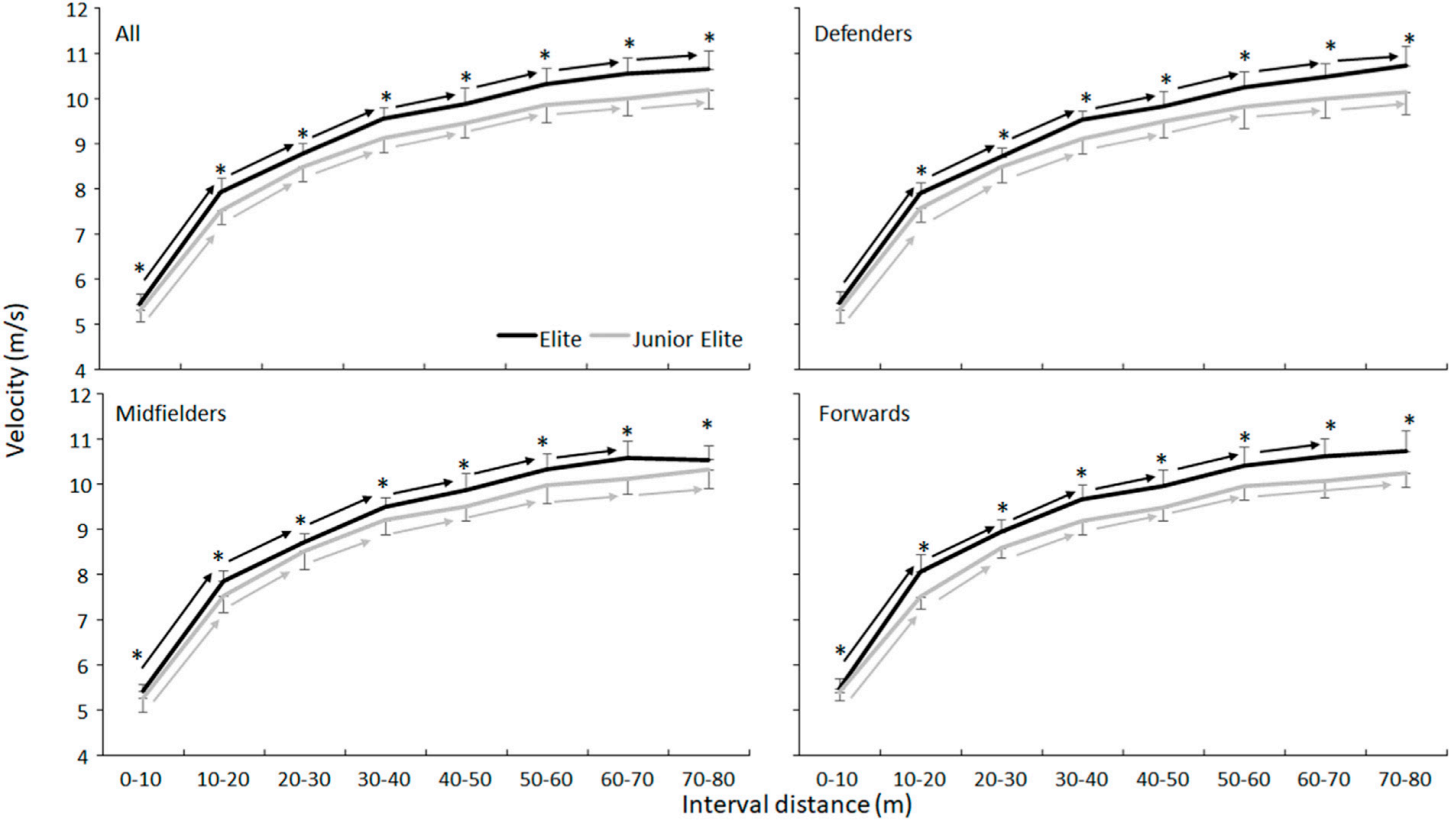
Mean velocity (SD) per 10 m averaged for all elite and junior players and per position in elite and junior players. * Indicates a significant difference between elite and junior players at this interval distance on a *p* < 0.05 level. → Indicates a significant increase in velocity between these two interval distances for this group on a *p* < 0.05 level.

When analysing the velocity over the different distances between level and position, a significant effect of velocity (F = 5479, *p* < 0.001, η_p_^2^ = 0.98), level (F = 20.3, *p* < 0.001, η_p_^2^ = 0.20), velocity x level (F = 4.4, *p* < 0.001, η_p_^2^ = 0.05) was found. No significant effects were found for position and other interaction effects (F ≤ 1.1, *p* ≥ 0.337, η_p_^2^≤0.03). Post hoc comparison showed that the elite players accelerated faster from 10 m upwards and reached a higher velocity (10.75 ± 0.37 vs. 10.24 ± 0.39 m/s) (Figure 1).

**FIGURE 2:**
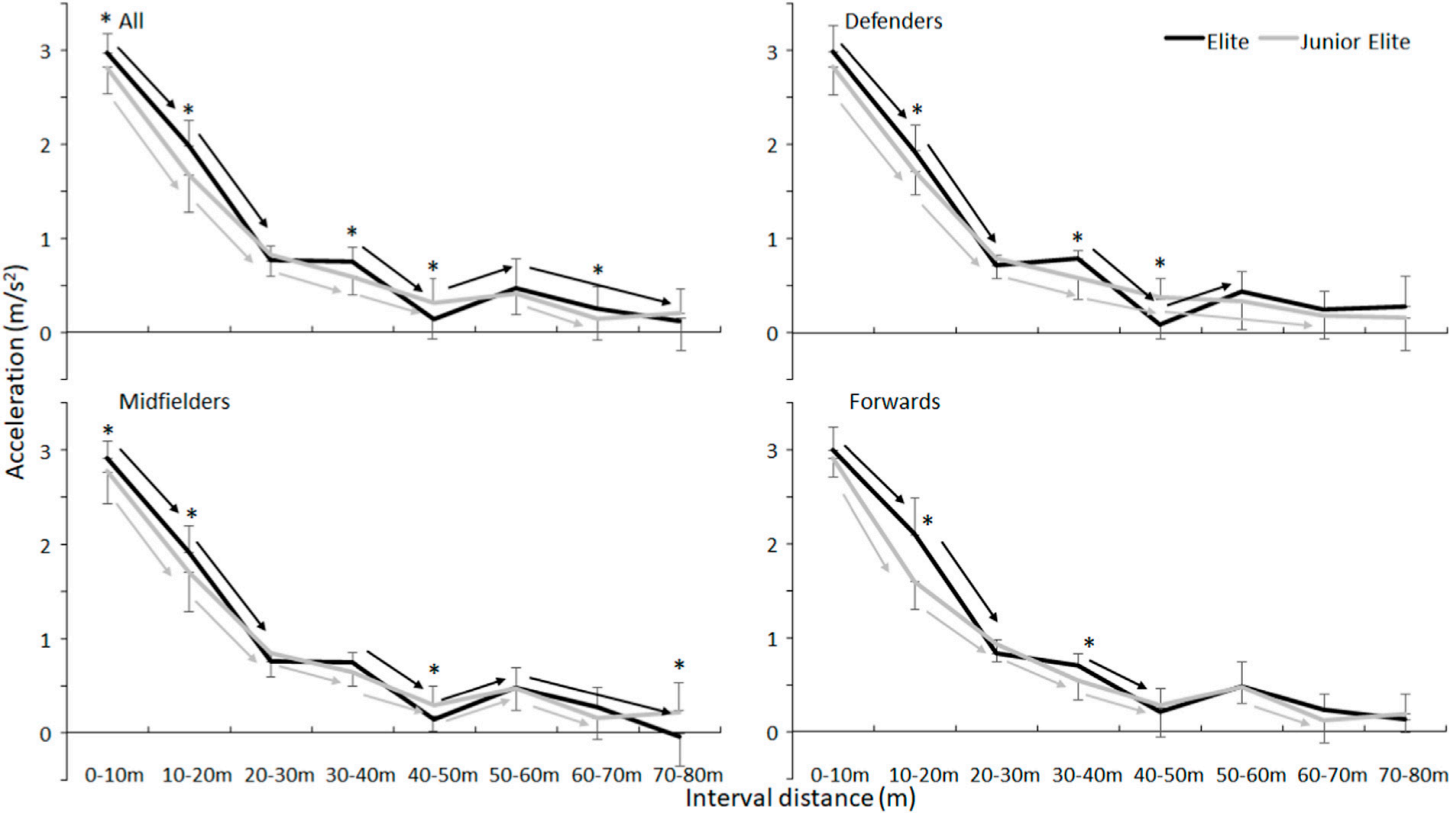
Mean acceleration (SD) per 10 m averaged for all elite and junior players and per position in elite and junior players. * Indicates a significant difference between elite and junior players at this interval distance on a *p* < 0.05 level. → Indicates a significant increase in acceleration between these two interval distances for this group on a *p* < 0.05 level.

Also, for acceleration at the different distances a significant effect for acceleration (F = 1066, *p* < 0.001, η_p_^2^ = 0.93), level (F = 19.8, *p* < 0.001, η_p_^2^ = 0.197) acceleration x level (F = 6.0, *p* < 0.001, η_p_^2^ = 0.069) was found. No significant effects were found for position and other interaction effects (F ≤ 0.7, *p* ≥ 0.499, η_p_^2^≤0.02). Post hoc comparison showed that the largest acceleration occurs the first 30 m in both groups, the elite players accelerated faster, especially the first 20 m and had the highest acceleration at the first 10 m (2.96 ± 0.22 vs. 2.81 ± 0.28 m/s^2^) (Figure 2).

## 4 Discussion

This study is the first to evaluate anthropometric and sprinting profiles of elite and junior (sub-elite) male bandy players with a specific focus on playing positions. The main findings were that no differences between positions were found in sprint skating performance (speed and acceleration), but that elite players were in general heavier than junior players and could accelerate faster, reaching a higher velocity over 80 m than the junior players. These findings were in line with those on female bandy players, which found similar results on position, level and skating performance and body mass differences (van den Tillaar et al., 2023).

The sprinting times are a bit faster than in similar ice hockey studies shown by a review of Stastny et al. (2023). The reason for this discrepancy was probably measurement of the first 10 m as the players started in the present study 0.5 m behind the first pair of photocells. However, this difference was around 0.25 s, which was similar to testing sprint performance when starting 0.5 m behind photo cells (Haugen et al., 2020). Thereby, the average velocity the first 10 m reached could be higher than those in ice hockey. However, in ice hockey the distances were in intervals of 7 m instead of 10 m. This resulted in similar velocities for the first 10 m in bandy with those of 7–14 m in ice hockey for elite players (5.44 ± 0.22 vs. 5.43 ± 0.53 m/s) as also acceleration was comparable (Figure 2). Yet, the maximal velocity reached in ice hockey was much lower than that in bandy (6.89 ± 0.44 vs. 10.66 ± 0.33 m/s), which was caused by the distance (ice rink) the players had to cover during the tests (40–48 m in ice hockey vs. 90 m in bandy). In that way the players in bandy were less limited by the distance to accelerate longer.

### 4.1 Playing level differences

In the present investigation, elite players could accelerate faster (Figure 2), reaching a higher velocity over 80 m than the junior players. This is in line with other studies that have demonstrated that elite players are faster than sub-elite players in bandy for women (van den Tillaar et al., 2023) and in ice hockey (Buckeridge et al., 2015; Peterson et al., 2015; Renaud et al., 2017; Vigh-Larsen et al., 2019). There are several possible explanations for these differences in acceleration and velocities between the levels. Firstly, due to more training experience elite players could have a better skating technique (e.g., larger range of motion and step width, greater stride rate and length), which enables them to optimally apply force in the push-off for propulsion and effective acceleration (Upjohn et al., 2008; Stull et al., 2011; Renaud et al., 2017; Shell et al., 2017). Secondly, more years of training including strength training elite players could have gained more muscle mass that enabled them to generate more power, which could inherently enable them to accelerate more quickly than junior elite players. It is known, that more muscularity bodies potentially enable athletes to exert force rapidly and apply it to the ground for maximal acceleration and speed (Miller, 2012). An indication for this is the higher body mass in elite players compared with the junior elite players.

Although no studies have examined the body mass and height differences between playing levels in bandy, the body mass and height of the senior players from the current study were in line with the results of previous studies that investigated only elite bandy players (Blomqvist et al., 2018; Johansson et al., 2021; Persson et al., 2021). Meanwhile, junior players from the current study were lighter than the elite bandy players from the earlier studies. However, in female bandy players also a lower body mass was found in junior players compared with the senior elite players (van den Tillaar et al., 2023). Because of similarities between ice hockey and bandy, we think that it is reasonable to compare the findings that have been obtained for players in these two on-ice sports.

In general, ice hockey players are heavier than bandy players (Buglione et al., 2013; Vigh-Larsen et al., 2019). This can be explained by several facts that differentiate the two sports. First, ice-hockey rules allow body checks, while they are not allowed in bandy. Therefore, heavier and stronger bodies can provide an advantage during collisions between ice-hockey players moving at high speeds. Second, heavier bodies could be more ineffective and energy consuming for bandy players who effectively play between 70 and 90 min, when compared to hockey players who are effectively engaged 15–25 min of multiple sprint bouts (Brocherie et al., 2018; Lignell et al., 2018; Persson et al., 2021). Although these facts differentiate ice hockey and bandy, studies of both male and female ice-hockey players have reported that elite players are heavier than sub-elite players (Buglione et al., 2013; Peterson et al., 2015; Renaud et al., 2017; Vigh-Larsen et al., 2019; Douglas et al., 2022), which is in line with our findings. Moreover, Vigh-Larsen et al. (2019) reported that elite players had a higher skeletal muscle mass and lower body fat percentage. Even though we did not examine fat free and fat mass, the differences in body mass could partially explain differences in sprinting performance.

### 4.2 Playing position differences

Even though there were no significant differences between playing positions in body height and mass and sprinting, the defenders and midfielders were slightly taller and heavier than the forwards in both playing level groups. In female bandy defence players were significantly heavier than forwards in both junior and senior elite players (van den Tillaar et al., 2023) but not taller. Unfortunately, no study has examined the differences between playing positions in bandy in men, neither for the body mass and height nor for the field-measurements of sprinting performance. However, in comparison to the research conducted recently in ice hockey, the results of the present study are in the line with Vigh-Larsen et al. (2019) and Jackson et al. (2017), who did not find any differences between defence and offense players from the Danish first and second divisions, and Canadian university players, respectively. In contrast, older studies by Vescovi et al. (2006) and Quinney et al. (2008) reported that defense players in the NHL were taller and heavier than offensive players. Considering that the rules of bandy do not allow body checks or intentional collisions between players, it is logical to assume that for bandy defensive players it is more important to get more quickly to the opponent forwards and win the ball than to use their bodies to collide, interrupt the opponent´s movement, and violate the rules. Thus, a lower body mass can provide them with optimal relative strength and power, which can enable them to efficiently overcome their body inertia and consequently move more quickly (Sekulic et al., 2021). Moreover, knowing that defenders have the highest workload playing the whole match without being substituted (Blomqvist et al., 2018), heavier bodies would be more energy consuming. In other words, a lower body mass, has an advantageous effect on aerobic capacity and endurance performance regardless of amount of body adiposity (e.g., normal or even low) or lean body mass (Maciejczyk et al., 2014). Consequently, this could be essential in long duration activities, dependent on aerobic capacity such as bandy match lasting 90 min.

In view of the recent motion-analyses in bandy, we assumed that offensive players, due to the specific game roles, would outperform defensive players in sprint skating performance. In brief, the offensive players (i.e., midfielders and forwards) spent more time in the fastest velocity zones (i.e., 20–25, 25–30 and >30 km/h) and above the lactate threshold than defensive players (i.e., libero and halves) (Blomqvist et al., 2018; Persson et al., 2021). This pattern is also seen during an ice-hockey match (Jackson et al., 2017; Douglas et al., 2019). However, Vigh-Larsen et al. (2019) and Jackson et al. (2017) reported no significant differences in the on-ice measurements (e.g., skating sprint and agility performance) between offensive and defensive players. It seems that the offensive players’ roles, which involve more time in fast velocity zones and high-intensity activities during the match play (Blomqvist et al., 2018; Persson et al., 2021), do not contributed to the advanced acceleration ability and fast skating that are measured in isolation outside the game. There are several potential explanations for the non-significant differences. First, we should not ignore the fact that we grouped players into three playing positions only, whereas in bandy there are five positions. Second, due to the lack of players, we pooled together senior and junior elite players, which could affect the power of the tests to discriminate the playing positions. Third, based on the studies in ice hockey that reported non-significant differences between playing positions in other conditioning capacities (e.g., power speed, strength, jump, flexibility) that are important for advanced skating performance (Vigh-Larsen et al., 2019), we can speculate that this was also the case in our sample. However, to confirm this speculation, more studies to investigate the fitness profile in bandy are needed.

### 4.3 Strengths and limitations

The strength of the present investigation is that we used a relatively large sample (n = 111) when compared to the other profiling studies that included only players from one bandy team (Blomqvist et al., 2018; Johansson et al., 2021; Persson et al., 2021). Moreover, this is the first study to have successfully implemented accurate on-ice measurement protocols to assess the acceleration and velocity profile in elite senior and junior bandy players. However, this study has several limitations that should be acknowledged. First, there is a risk that a smaller number of elite players and an unequal number of players in different playing positions could underestimate the power of the analyses. In addition, there were not enough goalkeepers to be included in the comparison. Furthermore, only the best skating attempt and not the average of both attempts was used for analysis. By using several attempts this perhaps could reduce measurement variability as indicated in other sprint (skating) studies (Simperingham et al., 2019; Stenroth et al., 2020; Perez et al., 2022b) and thereby result in other findings between positions and level. Moreover, the anthropometrics should have included the body composition measurements (e.g., fat free and fat mass percentage), and not only body height and mass. Additional biomechanical analyses are warranted to further explain the skating differences between playing levels.

## 5 Conclusion and practical application

In summary, there were no differences in the sprint skating performance between positions but playing level had a modulating effect upon acceleration capacity, with higher acceleration and velocity at the elite level when compared with the junior level. The coaches and professionals who work with young bandy players should be aware that the development of skating acceleration and speed might be of crucial importance if these players are to reach an elite playing level. This should include both the on- and off-ice strength, conditioning, and technical training. However, additional biomechanical and fitness profiling studies are needed to reveal the factors that contribute to playing level differences.

## Data Availability

The raw data supporting the conclusion of this article will be made available by the authors, without undue reservation.
